# A Case Report of a Candida auris Infection in Saudi Arabia

**DOI:** 10.7759/cureus.15240

**Published:** 2021-05-25

**Authors:** Mais B Alashqar, Lulwah Alabdan, Mohammad Khan, Abdul Hakim Almakadma, Sami Almustanyir

**Affiliations:** 1 College of Medicine, Alfaisal University College of Medicine, Riyadh, SAU; 2 Internal Medicine Department, Prince Mohammad Bin Abdulaziz Hospital, Riyadh, SAU; 3 Laboratory Department, Prince Mohammad Bin Abdulaziz Hospital, Riyadh, SAU; 4 Internal Medicine Department, Ministry of Health, Riyadh, SAU

**Keywords:** candidemia, candida auris, fungal infections, comorbidities, immunocompromised

## Abstract

*Candida auris* is a relatively new species of the *Candida* genus that is rapidly spreading in healthcare institutions across the globe. It is exceedingly difficult to identify with standard laboratory procedures and is challenging to treat due to its resistance to most antifungals. Moreover, it quickly colonizes on the surfaces in hospitals and ICUs and causes repeated infections, despite regular hospital disinfection. This grim occurrence of multidrug-resistant yeast has now become imperative to report, as its true prevalence remains unclear. Only some reports have been published in Saudi Arabia and here we present a case of *C. auris* candidemia identified in our hospital.

## Introduction

*Candida auris *is a novel type of opportunistic, nosocomial yeast pathogen that was first reported in 2009 in Japan [1]. Since its emergence, it has been identified in hospitals across five continents, particularly increasing in incidence during the COVID-19 pandemic [2,3]. This new species presents diagnostic challenges because it is difficult to identify with common microbiology procedures [2,4]. It is also difficult to treat as it is resistant to most antifungals [2,4]. There has been no consensus on the proper management of this infection and current protocols vary among healthcare centers.

The Centers for Disease Control and Prevention (CDC) has recommended that any identified cases be reported to local health authorities so that the spread of this multidrug-resistant yeast can be controlled [3,5].

## Case presentation

Our patient is a 48-year-old female from Riyadh, Saudi Arabia. She is married with three children, and her son provided the history during the clinical examination.

The patient presented to us with a four-day history of subjective fever, progressive shortness of breath, fatigue, and a dry cough. Her son also reported that she suffered two days of abdominal pain and diarrhea. Finally, she was unresponsive and drowsy. Systemic review was otherwise unremarkable.

Her past medical history included diabetes mellitus for five years, with an HbA1c of 7.5%, which is well controlled on metformin 750 mg once a day. She also had hypertension for the past five years, controlled on amlodipine 10 mg once a day. Finally, she is a known case of epilepsy with tonic-clonic seizures, for which she is taking levetiracetam 1,250 mg. She has had no previous surgeries.

Her family history was unremarkable. She is not a smoker and has no history of alcohol or drug abuse. She recently returned from a trip to Alshargyah district in Saudi Arabia after the Eid Holiday. She has no known allergies.

On presentation, the patient was lying in the bed, dyspneic, and unable to speak. Her temperature was 37.2 ˚C, heart rate was 82 beats per minute, respiratory rate 21 breaths per minute, blood pressure was 114/66, and oxygen saturation was 92% on room air.

Her Glasgow Coma Scale (GCS) was 8/15. She was aphasic, unable to move her limbs, unable to follow commands, but with spontaneous eye opening. Respiratory exam showed dyspnea at rest. On auscultation of the lungs, crackles were heard. Air entry and chest expansion were normal. Abdominal exam showed a soft, lax abdomen, with mild, diffuse tenderness. There was no distention or organomegaly noted.

Our patient was immediately intubated and then admitted to the ICU for observation and treatment. She was also tested for a COVID-19 infection, by reverse transcription-polymerase chain reaction (RT-PCR) of a nasopharyngeal swab specimen, which was found to be positive.

On the first day of her stay, she developed a low-grade fever of 37.8 ˚C. Her blood tests were notable for an elevated C-reactive protein of 4.16 mg/dL and a lactate dehydrogenase (LDH) of 295 U/L. After a chest X-ray was completed, she was diagnosed with a COVID-19 infection, complicated by pneumonia. Meropenem 500 mg and methylprednisolone 40 mg were, hence, started.

Her other inpatient medications included heparin 5,000 units (0.2 mL twice daily), levetiracetam 1250mg, Vitamin B1 100 mg, Vitamin C 500 mg, omeprazole 40 mg, rosuvastatin 20 mg, and lactulose 30 mL. She was also on potassium phosphate and sodium chloride IV infusions.

After the patient was given the prophylactic heparin, she developed hypotension and an expanding hematoma in the left thigh, measuring (11 cm x 2 cm), and therefore heparin was stopped.

Over the next two weeks, our patient had three more COVID-19 RT-PCR tests, which were all negative and eventually her pneumonia resolved. Nevertheless, she continued to struggle with maintaining adequate oxygenation, and so remained intubated until a tracheostomy was placed. Her GCS remained at 8/15.

Moreover, blood cultures revealed that the patient had a persistent *Acintobacter baumanni* bacteremia, for which she received IV imipenem/cilastatin (powder for injection 250 mg/250 mg) and vancomycin (1 g twice a day) for four weeks, but without improvement. This was then switched to imipenem and tigecycline (100 mg IV infusion, then 50 mg IV infusion twice a day) for 14 days.

**Figure 1:**
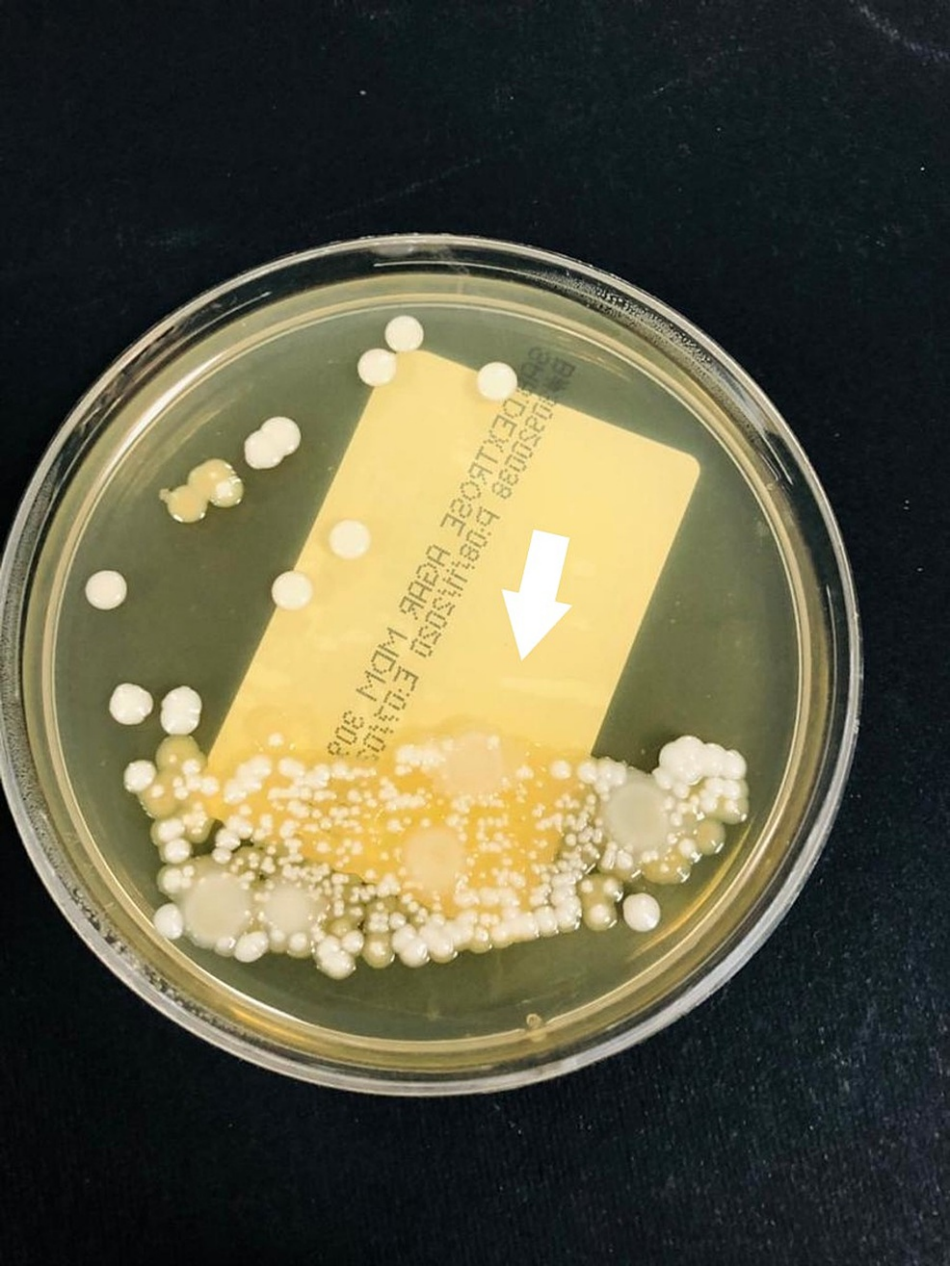
Candida auris colonies growing on Saboraux dextrose agar

**Figure 2:**
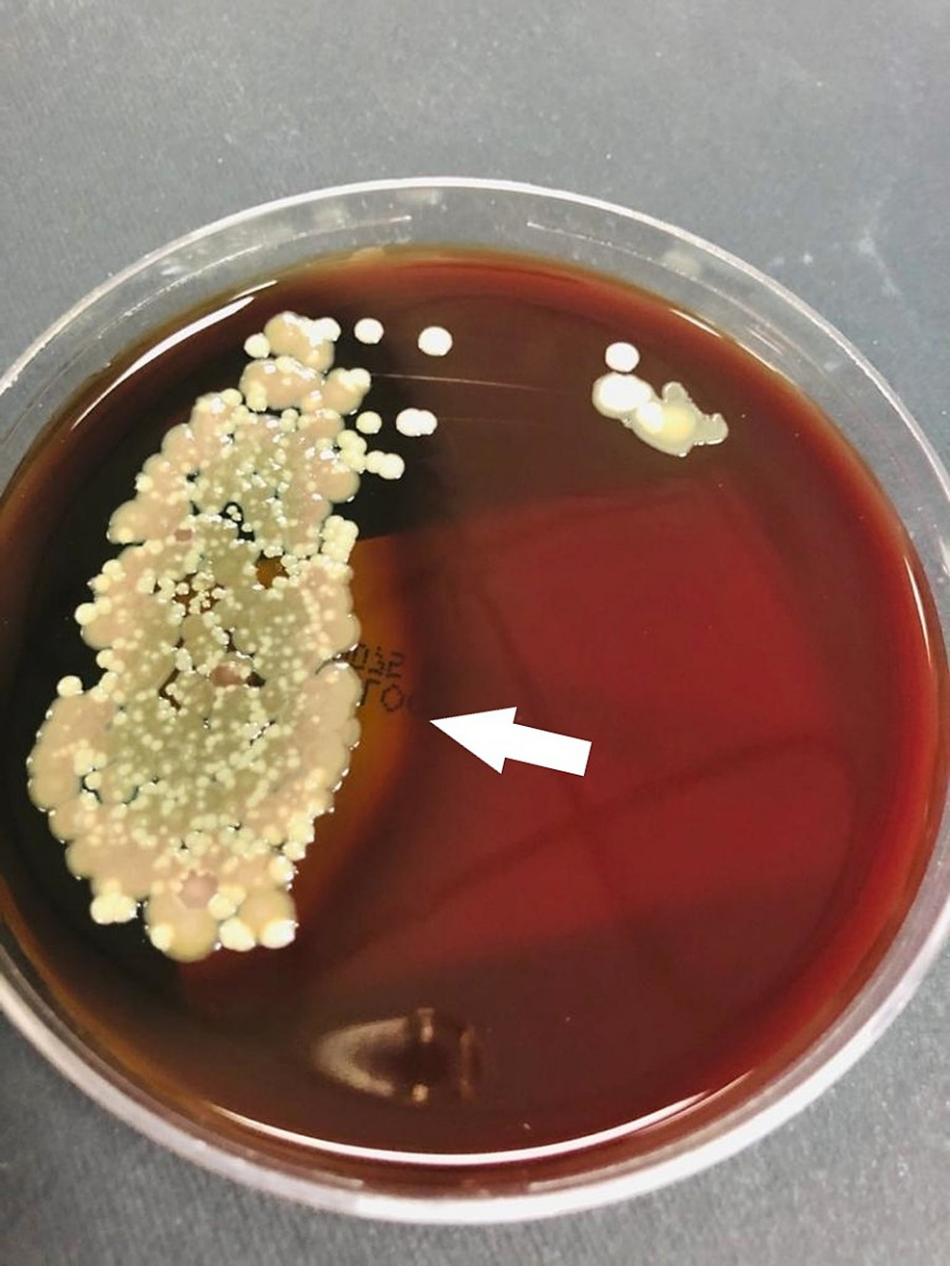
Candida auris colonies growing in sheep blood agar

On day 20 of admission, the lab results also indicated a *C. auris* candidemia, for which anidulafungin was started (loading dose was 200 mg IV, then 100 mg IV daily). The laboratory identification was based on VITEK-2 automated identification system (BioMerieux, France), and in the yeast culture, colonies grew at 42°C (Figures 1, 2). Despite treatment, however, the candidemia did not resolve.

During the third month of admission, our patient was still on a tracheostomy tube (to maintain an oxygen saturation above 95%), but with persistence of her candidemia. Unfortunately, since her neurological status did not improve, she was given a DNR (do-not-resuscitate) order. On day 90 of admission, she suffered sudden cardiorespiratory arrest, which was the cause of her decease.

Interestingly, during the two months following our patient’s admission, we isolated *C. auris* from two other ICU patients.

## Discussion

The *C. auris* species has been reported to have multiple clades based on the location where they were identified, namely: The South African, the South American, the South Asian, and the East Asian clades [2]. Many of the cases have been reported from India where one trauma center reported that among all cases of candidemia, *C. auris *was the second most common cause, accounting for 17.5% and exceeding *C. albicans* at their center [6].

This pathogen is identified most frequently in patients with multiple comorbidities, immunosuppression, patients who are admitted in ICUs, those who have been on ventilators, or those who have had recent surgeries (including central line insertions) [2,7,8]. These risk factors were present in our patient. Studies suggest that many patients had been on antibiotics and/or antifungals before the isolation of the *C. auris* species [2,7,8]. This infection has been found to cause local infections in the urinary tract, respiratory tract, ear canal, and around central line insertion sites [2,7,8]. It is, however, most frequently isolated from blood cultures in patients with candidemia [4].

The diagnostic dilemma with this infection is that common commercial identification systems used in microbiology laboratories for fungal identification cannot detect it reliably. These identification systems include the VITEK-2, BD Phoenix, and the API-20C [4]. It is most frequently misidentified as *C. haemulonii *but may also be mistaken for other fungal species as well [2]. Currently, the most reliable method of identification of *C. auris* is by Matrix Assisted Laser Desorption-ionization Time of light (Maldi TOF MS) (Bruker Inc., MA, USA), which utilizes a laser-based proteomic analysis of bacterial and fungal pathogens [9,10].

In fact, after the recognition of this species, a re-analysis of old samples in the SENTRY Antimicrobial Surveillance Program isolates collection was performed, and two misreported cases of *C. auris* were subsequently detected, from as early as 1996, making the actual prevalence of *C. auris* infections somewhat uncertain [2]. CDC reported the latest count of cases to be 1,708 in the United States, as of December 31, 2020 [5]. Furthermore, they reported that an additional 3,338 patients were colonized by *C. auris *across the country [5].

Most strains of this fungus are resistant to the triazole antifungals. Many are also resistant to amphotericin B, making the treatment of this infection problematic. There have been recommendations for using echinocandins, but resistance to this group has been increasing as well [2]. Micafungin has shown some success in a recent study conducted in mice [8].

Once introduced, this pathogen has a high tendency to colonize and spread quickly in a healthcare setting. Despite disinfection and preventative measures, this yeast can persist with multiple cases often occurring in the same hospital and/or the same ward [3,4].

Our case is in addition to others reported in Saudi Arabia. The first three cases were identified together in a study in 2018. Two of the three patients were successfully treated with amphotericin B and echinocandins, while the third passed away [9]. The fourth case was from the following year, but the isolates in his case were resistant to both antifungals. He, unfortunately, did not survive the aggressive infection [10] An outbreak of cases was reported in one center in 2020, with at least 34 cases identified, of which seven patients did not survive the infection [11]. Finally, two cases were described from a university hospital in Dammam city. Unfortunately, both patients passed away [12]. A study on the molecular characteristics of *C. auris* infections in Saudi Arabia confirmed that the isolates from the seven patients in their center were from the South Asian clade. These authors also corroborated the high rates of resistance to fluconazole and amphotericin B. They did mention, however, that none of their isolates were resistant to voriconazole, posaconazole, or echinocandins [13]. The slow, but sustained, spread of *C. auris *in Saudi Arabia is evident by the above-mentioned studies, as is the notable mortality associated with this infection.

## Conclusions

*C. auris *is an emerging threat in healthcare institutions, causing multiple types of hospital-acquired infections, with only a few treatment options. Definitive identification remains a challenge and more research is required to develop effective management guidelines when a *C. auris *infection is suspected in patients. More data are also needed to establish the best disinfection and safety protocols to prevent the colonization of this opportunistic pathogen in ICU units and hospital wards.
